# Chemical Composition and Phytotoxic and Antibiofilm Activity of the Essential Oils of *Eucalyptus bicostata*, *E. gigantea*, *E. intertexta*, *E. obliqua*, *E. pauciflora* and *E. tereticornis*

**DOI:** 10.3390/plants11223017

**Published:** 2022-11-08

**Authors:** Flavio Polito, Habiba Kouki, Sana Khedhri, Lamia Hamrouni, Yassine Mabrouk, Ismail Amri, Filomena Nazzaro, Florinda Fratianni, Vincenzo De Feo

**Affiliations:** 1 Department of Pharmacy, University of Salerno, Via San Giovanni Paolo II 132, 84084 Fisciano, Italy; 2 Laboratory of Biotechnology and Nuclear Technology, National Center of Nuclear Science and Technology, Sidi Thabet, B.P. 72, Ariana 2020, Tunisia; 3 Laboratory of Management and Valorization of Forest Resources, National Institute of Researches on Rural Engineering, Water and Forests, P.B. 10, Ariana 2080, Tunisia; 4 Institute of Food Science, CNR-ISA, Via Roma 64, 83100 Avellino, Italy

**Keywords:** *Eucalyptus*, essential oils, phytotoxicity, biofilm, metabolism inhibition

## Abstract

*Eucalyptus* species are characterized by their richness in essential oils (EOs) with a great diversity of biological activities. This study reports the chemical composition and the phytotoxic and antibiofilm activities of the EOs of six *Eucalyptus* species growing in Tunisia: *E. bicostata, E. gigantea, E. intertexta, E. obliqua, E. pauciflora* and *E. tereticornis*. Four EOs were rich above all in oxygenated monoterpenes (25.3–91.4%), with eucalyptol as the main constituent. However, in the EOs of *E. pauciflora* and *E. tereticornis,* sesquiterpene hydrocarbons (28.8–54.0%) were the main class of constituents; piperitone was the main constituent of both EOs. The phytotoxicity of the EOs was tested against germination and radicle elongation of the weeds *Sinapis arvensis* and *Lolium multiflorum* and the crop *Raphanus sativus*, resulting in the different inhibition of seed germination and radicle elongation depending on both chemical composition and the seed tested, with remarkable phytotoxicity towards *S. arvensis* and *R. sativus*. Furthermore, almost all EOs showed antibacterial potential, resulting in significant inhibition of bacterial biofilm formation and the metabolism of Gram-positive (*Staphylococcus aureus* subsp. *aureus* and *Listeria monocytogenes*) and Gram-negative (*Acinetobacter baumannii*, *Pseudomonas aeruginosa* and *Escherichia coli*) bacterial strains, in addition to acting on mature biofilms. The EOs were inhibitory against all bacterial strains tested and usually reluctant to undergo the action of conventional antibiotics. Therefore, these EOs may be considered for applications both as herbicides and in food and health fields.

## 1. Introduction

*Eucalyptus* is a genus of Myrtaceae, native to Australia and including about 900 species. The generic name is a word made up of the Greek terms “ευ,” which means “true,” and “καλψπτο,” which means to cover, referring to the calyx and corolla that form a coating that covers the flower until flowering. At the end of the 17th century, some of its species also began to be planted in Europe until they became widespread throughout the world over the centuries. The plants of this genus have many industrial uses, ranging from flexible and resistant wood for construction, to pulp for paper making, to honey obtained from flowers to rubber that flows along the bark. During the 1950s, 117 species of *Eucalyptus* were introduced in Tunisia, mainly intended to produce timber and fight against soil erosion [1].

However, only some species of this genus have been exploited to obtain essential oils (EOs), starting above all from the leaves, the uses of which are mainly in the pharmaceutical and cosmetic fields. These EOs are rich in monoterpenes and sesquiterpenes; other secondary metabolites are macrocarpals, alkaloids, phenols, flavonoids, tannins and phenolaldehydes [2]. The characteristic component of the volatile fractions of most *Eucalyptus* species is eucalyptol (1,8-cineole), whose content in the EO can reach up to 80–90% of the total [3]. Other main components are spatulenol, *p*-cymene, viridiflorol, α-phellandrene α-terpineol, limonene and α-pinene [4], along with α-, β- and γ-eudesmol, and piperitone [5]. Over the years, studies have been carried out to evaluate the possible biological activities of the EOs from leaves of *Eucalyptus* species. Traditional uses are oriented toward the treatment of various infectious diseases, flu, sore throat, cold, respiratory pathologies and painful states [6]. More recent studies have highlighted other biological activities not homogeneously distributed among the hundreds of species belonging to this genus. In particular, antimicrobial properties have been highlighted, mainly due to the presence of monoterpenes such as eucalyptol, α-pinene, β-pinene and limonene [7]. Additionally, phytotoxic and herbicidal activities on weeds and crops have been reported and attributed to EO components that can alter physiological and biochemical processes underlying the germination and elongation of roots [8]. For a long time, studies have been carried out on the allelopathic impact of the cultivation of some *Eucalyptus* species and of their metabolites (in particular monoterpenes) on both natural and agricultural ecosystems [9,10,11].

A bacterial biofilm constitutes a fairly complex structure made up of microbial cells associated with each other, which adhere to a surface, and are, in a certain sense, kept isolated from the external environment (although they are able to exert an important influence on it) through the formation of a sort of polysaccharide “dome” [12]. In the biomedical field, biofilms are involved in a wide range of diseases, such as joint and orthopedic diseases, and they also characterize a large number of chronic bacterial infections that have always been a major clinical problem, which can still be faced with many difficulties today. Bacterial biofilms are considered a serious hygiene problem in the environment, in human health and in the food industry. In fact, a biofilm makes bacteria much more resistant to disinfectants and to antimicrobial agents [13]. The cells contained in the biofilm are much more difficult to reach, and it becomes very difficult for synthetic drugs to “break” the organization of the biofilm due to how it is structured and composed. For this, there is a need to research and experiment with new types of substances that can make a fundamental contribution to the formation of biofilms and that are able to prevent their formation, persistence or even exacerbation after treatment. A promising way is that which analyzes how other organisms can defend themselves from bacterial colonization. Many plant organisms, for example, are continuously exposed to a wide range of potentially harmful microorganisms that can grow on their surfaces. Therefore, it is useful to study and understand the defense mechanisms that plants exploit to fight the microorganism. Many plant-derived compounds, especially EOs, have demonstrated anti-biofilm properties [14]. The chemical diversity among the countless plant species ensures an enormous reserve of substances that could make a fundamental contribution to the fight against biofilms, as well as creating mixtures of compounds to exploit multiple mechanisms at the same time.

The aim of this work was the study of the chemical composition of the EOs from six species of *Eucalyptus* grown in Tunisia, *E. bicostata* Maiden, Blakeley & Simmonds (=*E. globulus* subsp. *bicostata* (Maiden, Blakeley & Simmonds) J.B. Kirkp.), *E. pauciflora* Sieber ex Spreng., *E. gigantea* Hook f., *E. intertexta* R. T. Baker, *E. obliqua* L’Hér., and *E. tereticornis* Sm., and the valuation of their possible phytotoxic and antibiofilm activities. These species were chosen because they are well-adapted and acclimatized in Tunisia and have been poorly studied, both regarding the chemical composition of the EO and their biological activities.

## 2. Results

### 2.1. Composition of the EOs

The composition of the EOs is reported in Table 1 according to the elution order on a 5 HP column. The presence of 102 components distributed among the six species has been detected. The EO of *E. bicostata* showed the lowest number of components (19), with oxygenated monoterpenes (91.4%) as the main class. The other components are almost uniformly distributed among monoterpene hydrocarbons (1.7%), sesquiterpene hydrocarbons (1.1%), and oxygenated sesquiterpenes (2.5%). The main component was eucalyptol (85.5%); the other most representative components, whose concentration exceeded 1%, were α-pinene, *trans*-pinocarveol, pinocarvone and viridiflorol. This is the EO with the greatest percentage of eucalyptol among those analyzed. *E. gigantea* EO showed the presence of 36 components, with oxygenated monoterpenes (68.90%) as the main class. The main component was eucalyptol (59.3%), followed by spathulenol (11.8%) and α-terpineol (4.5%). Other components ranged from 0.1 to 2.0%; among these, α-eudesmol (2.0%), α-pinene (1.6%), *allo*-ocimene (1.5%), γ-terpinene (1.3%), terpinen-4-ol (1.3%) and α-epi-7-epi-5-eudesmol (1.0%) had a percentage higher than 1.0%. Forty-four components have been identified in the EO of *E. intertexta*, with a prevalence of oxygenated monoterpenes (75.0%). The main component was eucalyptol (65.9%), followed by spathulenol (8.1%), α-pinene (6.7%) and *trans*-pinocarveol (4.0%). The other components were present in very low quantities ranging from 0.1% to 1.2%, with cubebol (1,2%), pinocarvone (1,1%) and α-gurjunene (1,1%) with a percentage higher than 1.0%. The EO of *E. obliqua* revealed the greatest number of components (46), mostly oxygenated monoterpenes (68.6%) and small amounts of sesquiterpene hydrocarbons (0.7%). Eucalyptol was the main component (54.9%), followed by α-pinene (13.2%), spathulenol (3.8%), *trans*-pinocarveol (3.3%) and dihydrocarveol (3.2%). Other components in percentages higher than 1.0% were β-pinene (1.7%), *p*-cymene (1.4%), α-terpineol (1.2%) and globulol (1.2%). The EO of *E. pauciflora* showed the presence of 39 components distributed between monoterpene hydrocarbons (14.0%), oxygenated monoterpenes (25.5%), sesquiterpene hydrocarbons (28.8%), and oxygenated sesquiterpenes (27.1%). The most representative compounds were piperitenone and β-vetivenene (both 8.8%), followed by β-eudesmol (8.1%), *p*-cymene (7.6%), *trans*-dauca-4 (11), 7-diene (6.4%), γ-pathcoulene (6.3%) and α-eudesmol (6.3%). Eucalyptol resulted in only 2.0% of the total EO. In the EO of *E. tereticornis,* 33 components have been identified, primarily oxygenated sesquiterpenes (54.0%), followed by oxygenated monoterpenes (25.3%), oxygenated monoterpenes (11.4%) and monoterpene hydrocarbons (6.1%). The main component was piperitone (19.4%), followed by *trans*-dauca-4-(11),7-diene (17.9%), and β-vetivenene (17.3%). In this EO, eucalyptol was also present in a small amount (2.4%).

### 2.2. Phytotoxic Activity

Table 2, Table 3 and Table 4 report the phytotoxic activity of the EOs on *R. sativus*, *S. arvensis* and *L. multiflorum*, respectively. The results show a remarkable phytotoxic effect by the tested EOs, resulting in a dose-response inhibition of both germination and radical elongation. As regards *E. bicostata*, the inhibition of germination of *R. sativus* is high but never complete: the highest activity is at 1000 µg/mL (92.3% inhibition), while it is reduced at the other concentrations. However, it totally inhibited the germination of *S. arvensis* at concentrations of 1000 and 500 µg/mL, whereas at the lower concentrations tested, no appreciable activity was registered. This EO proved ineffective against *L. multiflorum*. The greatest inhibition on the radical elongation of *R. sativus* occurs at 1000 µg/mL (77.14%) and is slightly lower at 500 and 250 µg/mL (68.57 and 62.86%, respectively). The inhibitory activity on the elongation of *L. multiflorum* is instead low, exceeding 50% only at 1000 µg/mL (61.11%). *E. gigantea* EO showed a similar feature but with lower activity than *E. bicostata* EO. In fact, at 1000 µg/mL, this EO weakly inhibited the germination of *R. sativus* and *S. arvenis* (6.0 and 3.6%, respectively). It is unable to inhibit the germination of *L. multiflorum*. The inhibition of radical elongation against *R. sativus* is very low, being appreciable only at concentrations of 1000 (67.74%) and 500 µg/mL (54.84%). On the other hand, a higher inhibition was recorded against *S. arvensis* where, at all concentrations tested, it exceeds 80% with a maximum of 98.21% at 1000 µg/mL. Against *L. multiflorum*, the greatest activity occurs at 1000 µg/mL (88.24% inhibition). The *E. intertexta* EO inhibited the germination of *R. sativus* and *S. arvenis* at 1000 µg/mL, but the activity decays at lower concentrations, especially in the case of *S. arvensis*. This EO was unsatisfactory in inhibiting the germination of *L. multiflorum*. The inhibition activity of radical elongation was very high towards *R. sativus:* it resulted in total of 1000 µg/mL and of 80% at 500 µg/mL. The same activity is shown against *S. arvensis* where, however, there was appreciable activity even at 250 µg/mL (84.84%). Against *L. multiflorum*, this EO inhibited radical elongation by 88.64% at 1000 µg/mL. *E. obliqua* EO completely inhibited the germination of *S. arvensis* at concentrations of 1000 and 500 µg/mL while maintaining an appreciable activity at lower doses (16.7%). On the other hand, its action against *R. sativus* and *L. multiflorum* was unsatisfactory at all tested concentrations. The inhibitory activity on the radical elongation of *R. sativus* was 83.33% at 1000 µg/mL, while it did not reach 50% at other concentrations tested. The activity was instead very high against *S. arvensis,* where it was complete at 1000 and 500 µg/mL and achieved values of 80% at 250 and 72% at 125 µg/mL. Against *L. multiflorum*, the greatest activity occurred at 1000 (86.6%) and 500 µg/mL (70% inhibition). *E. pauciflora* EO showed significant activity against *R. sativus*, with 100% inhibition at 1000 µg/mL; at lower concentrations, the activity decayed. The activity was low towards *S. arvensis* and unsatisfactory towards *L. multiflorum*. The inhibition on radical elongation against *R. sativus* was total at 1000 µg/mL, while at other concentrations, it was not very noticeable. In the case of *S. arvensis*, the greatest activity occurred at 1000 µg/mL (81.08%), while the lower inhibition was registered at 500 µg/mL (56.76%). The concentrations of 125 and 250 µg/mL showed moderate activity (62.1 and 72.97%, respectively). In the case of *L. multiflorum,* the greatest inhibition occurred at 100 µg/mL (85.42%). *E. tereticornis* EO showed the most significant activity. At 1000 µg/mL, it totally inhibited the germination of all tested seeds, and at 500 µg/mL, it also completely inhibited the germination of *S. arvensis*. At lower doses, the activity decreased, especially against *L. multiflorum*. The inhibitory activity on the radical elongation of *R. sativus* is complete at 100 µg/mL. As regards *S. arvensis*, the inhibitory activity is complete at 1000 and 500 µg/mL and very high at 250 (93.33%) and 125 µg/mL (86.68%). In the case of *L. multiflorum*, the inhibitory activity was complete at 1000 µg/mL, but at other concentrations, it was not appreciable.

### 2.3. Antibacterial and Antibiofilm Activity

**Figure 1:**
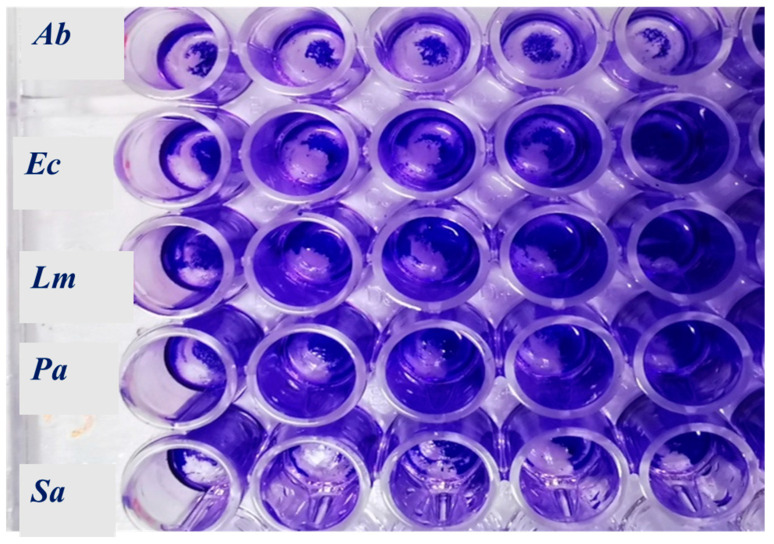
Typical bacterial biofilm after crystal violet staining and before dissolution with acetic acid 20%. *Ab: A. baumannii; Ec: E. coli; Lm: L. monocytogenes; Pa: P. aeruginosa; Sa: S. aureus*.

Table 5 shows the minimal inhibitory concentration of the six EOs necessary to impede the growth of the five pathogenic bacteria used as tester strains. Figure 1 shows typical bacterial biofilms of *A. baumannii, E. coli, L. monocytogenes, P. aeruginosa*, and *S. aureus*, formed in the 96-well microplates following staining with crystal violet. The capacity of the six EOs to fight bacterial adhesion and the process leading to mature bacterial biofilms is reported in Table 6. Table 7 reports the capacity of the EOs to work on the metabolism of the sessile cells, which can direct the bacterial cells to increase their virulence.

Except in a few cases and at the lowest concentration tested, all EOs proved capable of inhibiting biofilm formation by the five pathogenic bacterial strains, with inhibition rates as high as 85.12% (*E. gigantea* EO vs. *A. baumannii*) at the highest concentration used. This confirmed the biofilm inhibitory action observed for other *Eucalyptus* EOs, such as *E. gunnii* Hook. f., which demonstrated an effective inhibitory activity against some of the same strains used in our experiments, such as *S. aureus* and *E. coli*, albeit with greater inhibitory vigor, given the smaller amount of EO required to limit the bacterial biofilm. The results of the inhibitory activity on biofilm formation appeared attractive; those obtained concerning the mature biofilms, when the EOs were in contact with the bacterial strains after 24 h from the beginning of their growth, were still more interesting. A mature biofilm leads the bacteria to modify their morphological, metabolic, and physiological characteristics that generally determine their increased virulence. In our experiments, the tested EOs proved potentially helpful in limiting biofilm formation and acting against mature biofilms. In some cases, their inhibitory efficacy proved even more vigorous. For example, the EO of *E. bicostata* inhibited *A. baumannii* biofilm formation by 28.74% when tested at the lower concentration (10 µL/mL). Similar efficacy was also observed in the case of *S. aureus* and *L. monocytogenes*, against which *E. bicostata* EO practically showed similar efficacy on the mature biofilm. The action of the *E. bicostata* EO was powerful, indeed more decisive on the mature biofilm of *P. aeruginosa* (85.06% inhibition), compared to the inhibitory efficacy exerted by the same EO on the bacterial adhesion when at the lowest concentration tested, it was ineffective and, at 20 µL/mL, gave 58.74% inhibition. Similarly, the EO of *E. pauciflora*, which inhibited the *P. aeruginosa* biofilm formation by only 13.79%, proved to be much more effective on mature biofilms (47.97 and 56.20% inhibition at 10 and 20 µL/mL, respectively). Likewise, the EO of *E. obliqua* was more effective on the mature biofilm of *S. aureus* (77.38 and 83. 63% inhibition at 10 and 20 µL/mL, respectively) than on the adhesive process performed by this strain. Furthermore, although in the tests with the other strains, the inhibitory efficacy of the EOs was less pronounced, in each case, it was never insignificant, with the sole exception of the EO of *E. intertexta*, which was ineffective only against *L monocytogenes* at the lowest concentration tested. Through the MTT test, we also evaluated the effect that the two EO concentrations exerted on the sessile cell metabolism of the five bacterial strains with an upstream impact after adding the EO at time zero and on the mature biofilm. In the case of the MTT assay performed ab origine, the action of the EOs was, with some exceptions, mainly on bacterial metabolism. This was evidenced by the percent inhibition exhibited by the EOs of *E. bicostata*, *E. gigantea*, *E. obliqua*, and *E. pauciflora*. Some EOs, such as the EO of *E. gigantea*, had an intense inhibitory action on the metabolism of all pathogenic strains, with percentages of inhibition never less than 69.79% (10 µL/mL vs. *E. coli*). They went as high as 83.74% (*vs. A. baumannii*). The EOs of *E. bicostata* and *E. intertexta*, which failed to inhibit the metabolism of sessile *E. coli* cells, were the least effective. In other cases, some EOs, ineffective at the lowest concentrations, proved capable of acting on bacterial metabolism when tested at the highest concentration. Thus, in some cases, the inhibitory effect exerted by the EOs on the mature biofilm did not essentially translate to the metabolism of sessile cells. For example, the EO of *E. gigantea* was completely ineffective vs. *L. monocytogenes* and *P. aeruginosa*, and the EOs of *E. obliqua* and *E. pauciflora* did not act against *L. monocytogenes*. However, especially in the case of *E. pauciflora*, which also inhibited the mature biofilm of *L. monocytogenes* effectively, the inhibitory action did not translate to a step on the cellular metabolism but acted on its other characteristics, as amply demonstrated in the literature.

## 3. Discussion

**Figure 2:**
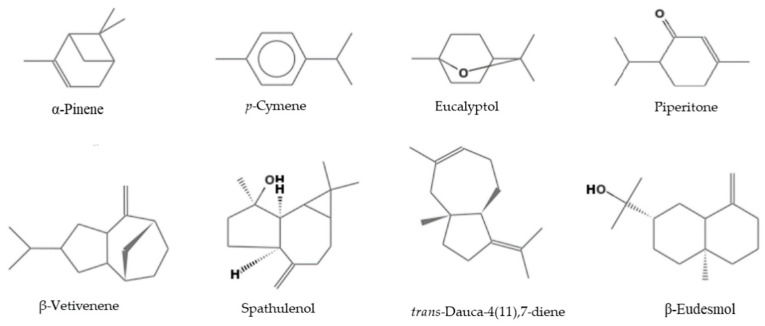
The main constituents of the EOs.

As shown in Table 8, the EO yields varied significantly between the species examined, from 0.03% for *E. tereticornis* to 3.11% for *E. obliqua*. These data agree with the yields in EOs found in this genus and with the considerable variability of percent composition found in the literature [15,16,17]. The EOs of *E. bicostata*, *E. gigantea*, *E. intertexta* and *E. obliqua* were characterized by the prevalence of monoterpenes (93.1, 74.7, 82.3, and 87.3%, respectively) with oxygenated monoterpenes as the main class (91.4, 68.9, 75.0, and 68.6%, respectively). Sesquiterpenes predominated in the EOs of *E. pauciflora* and *E. tereticornis* (55.9% and 65.4%, respectively), with hydrocarbons accounting for 28.8 and 54.0%, respectively. Eucalyptol was the main component in the EOs from *E. bicostata*, *E. gigantea*, *E. intertexta* and *E. obliqua*: this agrees with the literature, where this compound is reported as the main component of the EOs of most *Eucalyptus* species [1,6,15,16,17]. Instead, the EOs from *E. pauciflora* and *E. tereticornis* showed a much lower amount of eucalyptol. This agrees with the literature where these species are characterized by very low quantities of eucalyptol (up to a maximum of 20%) [4,15,16,18,19,20,21,22,23,24,25]. Figure 2 shows the main constituents of the EOs.

The composition of the EO of *E. bicostata* largely agrees with the literature on EOs obtained from plants of Tunisian origin [3,16], which report eucalyptol as the main component (in our sample in higher amounts), and the presence of α-pinene and *trans*-pinocarveol. Limonene and carvacrol are not present in our sample, while viridiflorol is present in a greater quantity, and globulol is present in smaller amounts. The composition of *E. gigantea* agrees with the studies of Elaissi and coworkers [1,15,16] on EOs obtained from Tunisian plants regarding the presence and percentage of eucalyptol but differs for the absence of limonene and *p*-cymene and the higher amount of spatulenol. The composition of the EO of *E. intertexta* agrees with data reported in the literature [25,26,27,28], where the main components were eucalyptol and *p*-cymene. However, in our sample, a greater number of components and considerable amounts of *trans*-pinocarveol and spatulenol were registered. Yong and coworkers [17] described the composition of an EO of *E. obliqua* of Australian origin. Our data agree with this study regarding the components and their percentages. The studies about the composition of the EO of *E. pauciflora* are inconsistent with our results. However, the common feature is the very low amount of eucalyptol [15,16,21,29,30,31]. The data regarding the composition of the EO of *E. tereticornis* disagree in part with those found in the literature [1,4,10,15,16,18,20,21,22,23,24]. However, the common feature was the low amount of eucalyptol and the significant presence of *p*-cymene and spathulenol. Our sample lacks limonene, cryptone, and caryophyllene oxide, previously reported as the main components. However, high amounts of sesquiterpenes are characteristic of our sample. To the best of our knowledge, the existence of chemotypes in the *Eucalyptus* genus has not yet been hypothesized. On the other hand, environmental conditions can significantly influence the composition of essential oils. The data collected may contribute to further studies that can investigate the diversity of chemical traits within the genus.

All the EOs have been shown to have a certain phytotoxic activity, which, however, is very variable according to the species and the seed considered: the most active EO in preventing the germination of *R. sativus* was the one obtained from *E. pauciflora*; the EO from *E. tereticornis*, on the other hand, is the most active in preventing the germination of *S. arvensis* and *L. multiflorum*. As for the inhibition of the radical elongation of *R. sativus*, the most active EO was the one obtained from *E. intertexta*; the EO of *E. teritecornis* was the most active in the inhibition of the radical elongation of *S. arvensis,* and the EO of *E. gigantea* was found to be the most active inhibitor of the radical elongation of *L. multiflorum*. In general, the phytotoxic activity was high towards seeds of *R. sativus* and *S. arvensis* and low towards *L. multiflorum*. For *E. bicostata*, *E. gigantea*, and *E. intertexta.* The phytotoxicity can be attributed to their high eucalyptol content [32]. However, the EOs of *E. gigantea* and *E. intertexta* showed a greater variety of compounds that can contribute together with eucalyptol to determine the total phytotoxic activity [33,34]. Different from the case of the EOs of *E. pauciflora* and mostly of *E. tereticornis*, with low amounts of eucalyptol, the most active on all three seeds. Therefore, their phytotoxicity was probably due to the synergism between the constituents [24,35]. The phytotoxic properties of the *Eucalyptus* genus are well recognized and reported [2,9], and the phytotoxic and allelopathic activities of some *Eucalyptus* species are well known and attributed to the EOs, in particular to monoterpenes such as eucalyptol and limonene [36], capable of acting in various ways, for example, destroying the chlorophyll reserves, interfering with cellular respiration processes [10] or decreasing the water reserves of the seeds [11]. Monoterpenes have been widely reported for their phytotoxic properties for a long time [37,38,39]. Sesquiterpenes have also been reported for their allelopathic properties [40]. In the examined EOs, the presence of components (both monoterpenes and sesquiterpenes) previously described as phytotoxic substances was registered [41,42]. Of importance seems the prevalence of eucalyptol in four of the analyzed EOs. This component is known for its phytotoxic properties [43,44], and it was proposed as a volatile inhibitor influencing the vegetation composition from the earliest studies on allelopathic interactions in *Salvia leucophylla* Greene populations [45]. Some mechanisms of action have been suggested [46], and eucalyptol has been proposed both for direct use as a bio-herbicide and as a lead compound for herbicide synthesis [47]. However, the EOs of *E. pauciflora* and *E. tereticornis* showed phytotoxicity even with low amounts of eucalyptol. Their activity can be, therefore, attributable to other components such as *p*-cymene, terpinen-4-ol, piperitone β-vetivenene, γ-patchoulene, α- and β-eudesmol, which are reported in the literature for their phytotoxic activity [10,24,38,48]. The antimicrobial activity of *Eucalyptus* EOs is well known [6,8,11,16,21]. The antibiofilm activity of the EOs studied is related to their composition: this property may be related to the presence of large amounts of eucalyptol, a compound with antibacterial effectiveness against several bacteria, which can act ab origine, limiting the initial steps of the biofilm formation [49], and, probably, also to a synergistic effect exerted by other constituents. In fact, the EOs with different compositions also showed the same effects: for example, *L. monocytogenes*, in which sessile metabolism in the mature biofilm was uninfluenced by the EOs of *E. pauciflora* and *E. bicostata*, although the two EOs contained 85.5% and 2.0% of eucalyptol, respectively. In any case, the EOs were inhibitory against Gram-positive and Gram-negative bacteria, usually reluctant to undergo the action of conventional antibiotics and can constitute natural products of interest to both the pharmaceutical and food sectors.

## 4. Materials and Methods

### 4.1. Plant Material

Leaves of the six *Eucalyptus* species were harvested from different Tunisian arboretums (Table 8). For each species, five samples from more than five different trees were collected and mixed for homogenization. The leaves were stored in a dry place for fifteen days. Specimens were identified at the National Institute of Research in Rural Engineering, Waters and Forests (INRGREF), Rue El Menzah, Tunis, Tunisia.

### 4.2. Extraction of the Essential Oils

One hundred grams of dried leaves of each species were submitted to hydrodistillation (500 mL of water) for 4 h using a Clevenger-type apparatus according to the method reported in the *European Pharmacopoeia* [50]. The EOs were solubilized in *n*-hexane, dried in an N_2_ atmosphere, and stored in amber vials in the refrigerator at 4 °C. Table 8 reports the data relating to the origin of plant material, with information about the collection site and the EO yields.

### 4.3. Analysis of the Essential Oils

The composition of the essential oils was examined by GC and GC-MS. GC analyses were performed using a Perkin-Elmer Sigma 115 gas chromatograph equipped with a flame ionization detector (FID) and a non-polar HP-5 MS capillary column of fused silica (30 m × 0.25 mm; 0.25 μm film thickness). The operating conditions were: injector and detector temperatures, 250 °C and 290 °C, respectively. The analysis was conducted on a scheduled basis: 5 min isothermally at 40 °C; subsequently, the temperature was increased by 2 °C/min until 270 °C, and finally, it was kept in an isothermal state for 20 min. The analysis was also performed on an HP Innowax column (50 m × 0.25 mm; 0.25 μm film thickness) using helium as a carrier gas (1.0 mL/min). GC-MS analysis was carried out through an Agilent 6850 Ser. II Apparatus equipped with a DB-5 fused silica capillary column (30 m × 0.25 mm; 0.25 μm film thickness) and connected to an Agilent Mass Selective Detector (MSD 5973) with an ionization voltage of 70 V and an ion multiplier energy of 2000 V. The mass spectra were scanned in the range of 40–500 amu, with five scans/s. The chromatographic conditions were as reported above; transfer line temperature was 295 °C. Most of the components were identified by comparing their Kovats indices (Ki) with those of the literature [51,52,53] and by a careful analysis of the mass spectra compared to those of pure compounds available in our laboratory or to those present in the NIST 02 and Wiley 257 mass libraries [54]. The Kovats indices were determined with a homologous series of *n*-alkanes (C_10_–C_35_) under the same operating conditions. For some components, the identification was confirmed through co-injection with standard compounds. The analyses were carried out in triplicate.

### 4.4. Phytotoxic Activity

To study the phytotoxic effects of the EOs on the seeds of *Raphanus sativus* L., *Sinapis arvenis* L., and *Lolium multiflorum* Lam., a bioassay based on germination and consequent radicle growth was used [55]. The seeds of *R. sativus* were purchased from Blumen srl, Piacenza, Italy; the seeds of *L. multiflorum* were purchased from the “Fratelli Ingegnoli” plant nursery, Milan, Italy, while the seeds of *S. arvensis* were collected from wild populations in Tunisia. The seeds were surface-sterilized with 95% ethanol for 15 s and sown in Petri dishes (Ø = 90 mm) containing five layers of Whatman filter paper impregnated with distilled water (7 mL, control) or a solution of the tested EO (7 mL). The EOs, solubilized in water:acetone (99.5:0.5), were tested at different doses of 1000, 500, 250, and 125 µg/mL using as controls water and a solution of water/acetone 99.5/0.5. Controls carried out with this solution did not differ from the control with water alone. A climatic chamber for growth was used, equipped with adjustable lighting, temperature, and humidity system. The germination conditions were 20 ± 1 °C with a natural photoperiod. The germination process was observed directly in the Petri dishes. A seed was considered germinated when root protrusion was evident [56]. After 120 h (fifth day), the effects on germination (the number of germinated seeds) and radicle elongation (measured in cm) were determined. Each determination was repeated 3 times using Petri dishes, each containing 10 seeds. The data were expressed as mean ± SD for germination and radicle elongation.

### 4.5. Antimicrobial Activity

#### 4.5.1. Microorganisms and Culture Conditions

*Acinetobacter baumannii* ATCC 19606, *Pseudomonas aeruginosa* DSM 50071, *Escherichia coli* DSM 8579 (Gram-negative), *Staphylococcus aureus* subsp. *aureus* Rosebach ATCC 25923 and *Listeria monocytogenes* ATCC 7644 (Gram-positive bacteria), used as bacterial test strains, were cultured for 18 h at 37 °C in Luria Broth at 80 rpm (Corning LSE, Pisa, Italy). *A. baumannii* was cultured at 35 °C under the same conditions.

#### 4.5.2. Minimal Inhibitory Concentration (MIC)

The MICs of the EOs were evaluated in flat-bottomed 96-well microtiter plates, which were incubated at 37 °C (35 °C for *A. baumannii*) for 24 h. The value of the MIC was revealed through the color change occurring from dark purple to colorless [57]. Tetracycline (μg/mL) was used as a positive control.

#### 4.5.3. Biofilm Inhibitory Activity

The ability of the EOs to affect bacterial adhesion was investigated following the method of Fratianni and coworkers [58] with flat-bottomed 96-well microtiter plates. Before the test, the bacterial cultures were adjusted to 0.5 McFarland with fresh culture broth. Then, 10 µL of the bacterial cultures and 10 or 20 μL/mL of the EOs were placed in each well, and the wells were filled with different volumes of Luria–Bertani broth to reach a final volume of 250 µL/well. Plates were covered with parafilm tape to avoid evaporation and incubated for 48 h at 37 °C (35 °C for *A. baumannii*). After the removal of the planktonic cells, sessile cells were washed twice with a sterile physiological solution, which was removed. The plates were left for 10 min under a laminar flow hood. Two hundred µL of methyl alcohol were added to each well to fix the sessile cells and removed after 15 min. Each plate was left to let the dryness of the samples. Two hundred µL of 2% *w/v* crystal violet solution/well was used for 20 min to stain the sessile cells. Plates were washed with a sterile physiological solution and left to dry. The bound dye’s release was obtained by adding 200 µL of glacial acetic acid 20% *w/v*. The absorbance was assessed at 540 nm (Cary Varian, Milano, Italy). The percent value of adhesion was calculated with respect to the control (formed by the cells grown without the presence of the samples, inhibition rate of 0%). Triplicate tests were performed, taking into account the average results for reproducibility.

#### 4.5.4. Activity on Mature Bacterial Biofilm

The overnight bacterial cultures were adjusted to 0.5 McFarland with fresh Luria–Bertani culture broth, and 10 μL were added to flat-bottomed 96-well microtiter plates to have a final volume of 250 μL/well. Next, microplates were covered with parafilm tape to avoid evaporation and incubated at 37 °C (35 °C for *A. baumannii*). After 24 h of bacterial growth, planktonic cells were removed, and the EOs (10 or 20 μL/mL) and Luria–Bertani broth were added to have a final volume of 250 μL/well. After 24 h of incubation, the sequential steps of the experiment, including the calculation of the percent value of inhibition compared with the untreated bacteria, were performed as previously described.

#### 4.5.5. Effects of EOs on Cell Metabolic Activity within the Biofilm

The effect on the metabolic activity of the bacterial cells of two concentrations (10 or 20 μL/mL) of the EOs, which were added at the beginning of the bacterial growth and after 24 h of incubation, was investigated through the 3-(4,5-dimethylthiazol-2-yl)-2,5-diphenyltetrazolium bromide (MTT) colorimetric method [59]. After a 48 h incubation period, planktonic cells were discarded; 150 μL of sterile physiological solution and 30 μL of 0.3% MTT (Sigma, Milano, Italy) were added. Microplates were kept at 37 °C (35 °C for *A. baumannii*). After two h, the MTT solution was removed, and two washing steps were performed with 200 μL of sterile physiological solution. Then, 200 μL of dimethyl sulfoxide (DMSO, Sigma, Milano, Italy) was added to allow the dissolution of the formazan crystals, measured after 2 h at 570 nm (Cary Varian, Milano, Italy).

### 4.6. Statistical Analysis

Results were expressed as mean ± standard deviation (SD) of three independent experiments and analyzed by one-way analysis of variance (ANOVA) by GraphPad Prism 6.0 (Software Inc, San Diego, CA, USA). Results were considered significant for *p* < 0.05.

## 5. Conclusions

The data collected in this study on the chemical composition of the EOs of the studied species help to shed light on the complex phytochemistry of EOs of the *Eucalyptus* genus, even if grown outside its habitat. Moreover, the phytotoxic activity of the studied EOs can be exploited to obtain selective herbicides on target species. EOs in agriculture have many advantages: they come from plant organisms already present in nature and therefore are characterized by high eco-compatibility. Their use is considered a safe strategy in crop management systems in the context of the circular economy and respect for the environment. EOs are a valid alternative to control pathogens, agricultural pests, and weeds, thus avoiding the indiscriminate use of agrochemicals that negatively influence the environment and human health. The activity of the studied EOs against the pathogenic bacteria tested could be considered of noticeable significance in different fields of application. In recent years, the increase in some infections has been linked to the expansion of the presence in several environments of the strains used in our experiments. Such bacteria have developed a more robust evolutionary drug resistance due to different factors, including inappropriate use of conventional drugs if not required or indispensable. Thus, the interest in natural alternatives to prevent biofilm formation and fight mature biofilms, which are more challenging to eradicate, augmented the research to identify natural agents as alternatives to conventional sanitizers to control biofilm development by acting on bacteria metabolism and/or other bacterial cell parameters. Under such a point the view, the six EOs demonstrated an important role in fighting the bacterial biofilm both at the beginning of the biofilm formation process and at the mature stage, which is the most ideal situation for bacteria, which, protected by the biofilm niches, become more protected and less sensitive to the action of conventional drugs. Finally, a suggestive working hypothesis could orient future research toward a possible link between phytotoxic and antibacterial activities.

## Figures and Tables

**Table 1:** Chemical composition (%) of the EOs.

	Compound Name	*E. bicostata*	*E. gigantea*	*E. intertexa*	*E. obliqua*	*E. pauciflora*	*E. tereticornis*	Ki ^a^	Ki ^b^	Identification ^c^
1	α-Pinene	1.7 ± 0.1	1.6 ± 0.1	6.7 ± 0.3	13.2 ± 0.4	0.2 ± 0.0	-	867	1012	1,2,3
2	Camphene	t	-	0.1 ± 0.0	0.2 ± 0.0	-	-	876	1075	1,2,3
3	β-Pinene	t	0.3 ± 0.0	0.2 ± 0.1	1.7 ± 0.2	0.1 ± 0.1	-	902	1110	1,2,3
4	α-Phellandrene	-	-	0.1 ± 0.1	0.7 ± 0.1	2.2 ± 0.2	1.1 ± 0.2	930	1177	1,2,3
5	α-Terpinene	-	0.1 ± 0.0	-	0.1 ± 0.0	0.7 ± 0.2	0.2± 0.0	942	1170	1,2,3
6	*p*-Cymene	-	-	-	1.4 0 ± 01	7.6 ± 0.3	4.6 ± 0.3	952	1250	1,2,3
7	β-Phellandrene	-	-	-	-	2.8 ± 0.2	-	954	1189	1,2,3
8	Eucalyptol	85.5 ± 0.6	59.3 ± 0.5	65.9 ± 0.5	54.9 ± 0.5	2.0 ± 0.0	2.4 ± 0.1	958	1210	1,2,3
9	(E)-β-Ocimene	-	0.5 ± 0.0	-	-	-	-	968	1242	1,2,3
10	(Z)-β-Ocimene	-	-	-	-	0.1 ± 0.0	-	977	-	1,2,3
11	*p*-Mentha-2,4(8)-diene	-	-	-	0.8 ± 0.0	-	-	983	-	1,2
12	γ-Terpinene	-	1.3 ± 0.1-	-	-	0.1 ± 0.0	-	984	1221	1,2,3
13	*cis*-Sabinenehydrate	-	0.2 ± 0.0	-	-	-	-	997	1115	1,2
14	Terpinolene	-	0.5 ± 0.1	0.1 ± 0.0	0.3 ± 0.0	0.3 ± 0.0	0.2 ± 0.0	1008	1267	1,2,3
15	6-Camphenone	-	-	0.1 ± 0.0	-	-	-	1011	-	1,2
16	Linalool	-	0.5 ± 0.1	-	-	-	-	1024	1506	1,2,3
17	*endo*-Fenchol	-	-	-	-	4.4 ± 0.5	-	1025	-	1,2
18	3-Methylbutyl 3-methylbutanoate	-	-	0.2 ± 0.0	0.2 ± 0.0	-	-	1029	1285	1,2
19	*exo*-Fenchol	0.1	-	0.3 ± 0.0	0.4 ± 0.0	-	-	1031	1591	1,2
20	*trans*-*p*-Mentha-2,8-dien-1-ol	-	-	-	0.1 ± 0.0	-	-	1040	1639	1,2
21	*cis*-*p*-Menth-2-en-1-ol	-	-	-	-	2.9 ± 0.6	0.2 ± 0.0	1041	-	1,2
22	α-Campholenal	-		0.1 ± 0.0	0.2 ± 0.0	-	-	1043	1485	1,2
23	*allo*-Ocimene	-	1.5 ± 0.0	0.1 ± 0.2	0.3 ± 0.0	-	-	1051	1388	1,2,3
24	*trans*-Pinocarveol	2.5± 0.2	0.5± 0.1	4.0 ± 0.2	3.3 ± 0.2	-	0.1 ± 0.0	1057	1664	1,2
25	*cis*-β-Terpineol	-	-	-	-	-	0.1 ± 0.0	1058	-	1,2
26	*cis*-Verbenol	-	-	-	0.4 ± 0.0	-	-	1058	1665	1,2
27	Camphor	-	-	0.1 ± 0.0	-	-	-	1059	1491	1,2,3
28	Citronellal	-	-	0.1 ± 0.0	-	-	-	1063	1487	1,2
29	Sabina ketone	-	0.4 ± 0.0	0.1 ± 0.0	0.5 ± 0.0	-	-	1067	1651	1,2
30	*trans*-Pinocamphone	-	-	t	0.1 ± 0.0	-	-	1074	-	1,2
31	Pinocarvone	1.6 ± 0.2	-	1.1 ± 0.1	0.7 ± 0.0	-	-	1077	1586	1,2
32	Borneol	0.2 ± 0.0	0.4 ± 0.0	0.3 ± 0.0	0.6 ± 0.0	-	-	1082	1715	1,2,3
33	*p*-Mentha-1,5-dien-8-ol	-	-	0.2 ± 0.0	-	-	-	1087	1670	1,2
34	Terpinen-4-ol	-	1.3 ± 0.1	0.5 ± 0.0	-	3.1 ± 02	1.7 ± 0.2	1095	1590	1,2,3
35	(E)-*iso*-Citral	-	-	-	-	0.6 ± 0.0	0.9 ± 0.0	1099	-	1,2
36	*cis*-Pinocarveol	-	-	0.3 ± 0.0	0.2 ± 0.0	-	-	1099	-	1,2
37	*trans*-Isocarveol	0.4 ± 0.0	-	-	-	-	-	1099	1810	1,2
38	*cis*-Dihydrocarvone	-	-	-	0.8 ± 0.1	-	0.2 ± 0.0	1101	-	1,2
39	Dihydrocarveol	0.5 ± 0.0	-	0.2 ± 0.0	3.2 ± 0.2	-	-	1102	-	1,2
40	Cryptone	-	0.4 ± 0.0	-	-	-	-	1103	1659	1,2
41	*cis*-Piperitol	-	-	-	-	1.1 ± 0.0	-	1106	1758	1,2
42	Myrtenol	0.2 ± 0.0	-	0.3 ± 0.0	-	-	-	1107	1791	1,2
43	α-Terpineol	0.1 ± 0.0	4.5 ± 0.2	0.9 ± 0.1	1.2 ± 0.1	1.1 ± 0.1	0.3 ± 0.0	1110	1661	1,2,3
44	Safranal	-	-	-	0.3 ± 0.0	-	-	1117	1648	1,2
45	*trans*-Piperitol	-	-	-	-	1.5 ± 0.1	-	1120	1690	1,2
46	*cis*-4-Caranone	-	-	-	0.1 ± 0.0	-	-	1134	-	1,2
47	*cis*-Carveol	-	-	0.1 ± 0.0	-	-	-	1135	1848	1,2
48	Verbenone	-	-	-	0.2 ± 0.0	-	-	1143	1726	1,2
49	*cis*-*p*-Mentha-1(7),8-dien-2-ol	0.3 ± 0.0	0.2 ± 0.0	0.3 ± 0.0	-	-	-	1144	1896	1,2
50	Cuminaldehyde	-	-	-	0.4 ± 0.0	-	-	1149	1753	1,2
51	Carvone	-	-	-	0.2 ± 0.0	-	-	1156	1736	1,2
52	*exo*-Fenchyl acetate	-	-	0.2 ± 0.0	-	-	-	1158	-	1,2
53	Piperitone	-	0.9 ± 0.1	-	0.1 ± 0.0	8.8 ± 0.3	19.4 ± 0.5	1166	1748	1,2
54	α-Terpinen-7-al	-	0.4 ± 0.0	-	-	-	-	1198	1811	1,2
55	Thymol	-	0.3 ± 0.0	-	0.7 ± 0.1	-	-	1218	2172	1,2,3
56	γ-Terpinen-7-al	-	-	-	0.1 ± 0.0	-	-	1236	-	1,2
57	δ-Elemene	-	-	-	-	0.7 ± 0.1	2.2 ± 0.1	1237	1479	1,2,3
58	*trans*-Verbenyl acetate	-	-	-	0.2 ± 0.0	-	-	1244	-	1,2
59	6-camphenol acetate	-	-	0.1 ± 0.0	-	-	-	1245	-	1,2
60	*p*-Menth-1-en-9-ol	-	-	-	0.4 ± 0.0	-	-	1252	-	1,2
61	Copaene	-	0.1 ± 0.0	-	-	-	-	1265	1477	1,2,3
62	α-Cubebene	-	-	-	-	-	0.1 ± 0.0	1270	1442	1,2
63	β-Elemene	-	-	-	-	0.1 ± 0.0	0.2 ± 0.0	1290	-	1,2,3
64	β-Longipinene	-	-	-	-	0.1 ± 0.0	0.3 ± 0.0	1298	-	1,2
65	α-Gurjunene	-	0.5 ± 0.0	1.1 ± 0.1	-	-	-	1300	1535	1,2
66	α-Caryophyllene	0.1 ± 0.0	-	-	-	-	-	1307	1617	1,2
67	(Z)-Caryophyllene	-	-	0.2 ± 0.0	-	-	-	1308	1617	1,2
68	Germacrene D	-	-	-	0.2 ± 0.0	-	-	1327	1712	1,2
69	Longifolene	-	-	-	-	0.8 ± 0.1	2.6 ± 0.2	1328	1574	1,2
70	Aromadendrene	0.8 ± 0.0	-	0.2± 0.0	-	0.6 ± 0.0	2.9 ± 0.3	1348	1631	1,2
71	*allo*-Aromadendrene	0.2 ± 0.0	-	-	-	0.1 ± 0.0	-	1349	1660	1,2
72	(E)-Caryophyllene	-	0.2 ± 0.0	-	-	-	-	1355	1612	1,2
73	α-Himachalene	-	-	-	-	0.3 ± 0.0	-	1366	-	1,2
74	9-*epi*-(E)-Caryophyllene	-	0.7	-	-	0.4 ± 0.0	0.8 ± 0.1	1376	-	1,2
75	*cis*-β-Guaiene	-	-	-	0.3 ± 0.0	-	-	1383	-	1,2
76	γ-Gurjunene	-	0.1 ± 0.0	0.4 ± 0.0	-	1.2 ± 0.1	2.8 ± 0.2	1384	-	1,2
77	α-Vetispirene	-	-	-	-	-	0.4 ± 0.0	1401	-	1,2
78	γ-Amorphene	-	-	-	-	-	0.1	1408	-	1,2
79	*epi*-Cubebol	-	0.8 ± 0.0	-	0.2 ± 0.0	-	-	1426	1957	1,2
80	γ-Patchoulene	-	-	-	-	6.3 ± 0.2	4.2 ± 0.2	1438	-	1,2
81	Cubebol	-	-	1.2 ± 0.1	-	-	-	1441	-	1,2
82	Viridiflorene	-	0.7 ± 0.0	0.1 ± 0.0	0.2 ± 0.0	0.2 ± 0.0	1.2 ± 0.1	1448	-	1,2
83	*trans*-β-Guaiene	-	-	0.5 ± 0.0	-	2.8 ± 0.3	1.0 ± 0.0	1449	-	1,2
84	β-Vetivenene	-	-	-	-	8.8	17.3	1463	-	1,2
85	Viridiflorol	1.8	-	-	0.4 ± 0.0	-	-	1464	2110	1,2
86	Globulol	0.4	-	0.9 ± 0.0	1.2 ± 0.0	-	-	1466	2104	1,2
87	Spathulenol	-	11.8 ± 0.5	8.1 ± 0.3	3.8 ± 0.3	-	-	1468	2127	1,2
88	Cubeban-11-ol	-	0.5 ± 0.0	0.3 ± 0.0	0.2 ± 0.0	-	-	1476	-	1,2
89	*trans*-Dauca-4(11),7-diene	-	-	-	-	6.4 ± 0.1	17.9 ± 0.3	1477	-	1,2
90	Guaiol	-	-	-	-	2.5 ± 0.1	-	1478	2094	1,2
91	Rosifoliol	-	-	0.5 ± 0.0	0.5 ± 0.0	-	-	1483	-	1,2
92	α-*epi*-7-epi-5-Eudesmol	-	1.0 ± 0.0	-	-	2.7 ± 0.1	4.2 ± 0.1	1485	-	1,2
93	*allo*-Aromadendreneepoxide	-	-	0.2 ± 0.0	-	-	-	1496	-	1,2
94	*epi*-Cedrol		0.4 ± 0.0	-	-	1.5 ± 0.1	2.6 ± 0.1	1497	-	1,2
95	α-Cadinol	-	-	0.2 ± 0.0	-	-	-	1502	2224	1,2
96	γ-Eudesmol	-	0.4 ± 0.0	-	0.5 ± 0.0	4.7 ± 0.1	2.0 ± 0.1	1508	2178	1,2
97	14-hydroxy-(Z)-Caryophyllene	-	-	0.1 ± 0.0	-	-	-	1513	-	1,2
98	*cis*-Cadin-4-en-7-ol	-	0.8 ± 0.0	-	-	1.3 ± 0.1	-	1515	-	1,2
99	β-Eudesmol	0.3	0.8 ± 0.0	0.4 ± 0.0	-	8.1 ± 0.5	2.2 ± 0.1	1527	2248	1,2
100	α-Eudesmol	-	2.0 ± 0.0	0.3 ± 0.0	0.3 ± 0.0	6.3 ± 0.4	-	1530	2247	1,2
101	5-Hydroxy-isobornyl isobutanoate	-	0.1 ± 0.0	-	0.1 ± 0.0	-	-	1540	-	1,2
102	Vulgarone B	-	-	-	-	-	0.4 ± 0.0	1543	-	1,2
	Total	96.7	96.0	97.4	96.1	95.5	96.8			
	Monoterpene hydrocarbons	1.7	5.8	7.3	18.7	14.0	6.1			
	Oxygenated monoterpenes	91.4	68.9	75.0	68.6	25.5	25.3			
	Sesquiterpene hydrocarbons	1.1	2.3	2.5	0.7	28.8	54.0			
	Oxygenated sesquiterpenes	2.5	18.5	12.2	7.1	27.1	11.4			

^a,b^ The Kovats retention indices determined relative to a series of *n*-alkanes (C10–C35) on the apolar HP-5 MS and the polar HP Innowax capillary columns, respectively. ^c^ Identification method: 1 = comparison of the Kovats retention indices with published data, 2 = comparison of mass spectra with those listed in the NIST 02 and Wiley 275 libraries and with published data, and 3 = co-injection with authentic compounds; t = trace (<0.1%). - = absent.

**Table 2:** Phytotoxic activity of the EOs on *R. sativus*.

**Germinated Seeds**						
	***E. bicostata***	***E. gigantea***	***E. intertexta***	***E. obliqua***	***E.pauciflora***	***E. tereticornis***
Control (H_2_O)	8.7 ± 1.2	9.7 ± 0.6	8.3 ±0.6	10.0 ± 0.0	9.3 ± 0.6	9.9 ± 3.5
125 µg/mL	2.0 ± 1.0 ****	5.7 ± 0.6 ****	4.0 ± 1.7 *	8.3 ± 1.2	6.7 ± 1.5 ****	6.9 ± 1.5
250 µg/mL	1.7 ± 2.1 ****	4.7 ± 0.6 ****	6.3 ± 1.5	7.7 ± 0.6 *	4.7 ± 0.6 ****	3.0 ± 1.0
500 µg/mL	1.0 ± 1.0 ****	1.3 ± 0.6 ****	3.0 ± 2.0 ***	7.3 ± 1.2 *	1.3 ±0.6 ****	3.0 ± 1.0
1000 µg/mL	0.7 ± 0.6 ****	0.7 ± 0.6 ****	0.0 ± 0.0 ****	6.0 ± 1.0 ****	0.0 ± 0.0 ****	0.0 ± 0.0 *
**Radical Length (cm)**						
	***E. bicostata***	***E. gigantea***	***E. intertexta***	***E. obliqua***	***E.pauciflora***	***E. tereticornis***
Control (H_2_O)	3.5 ± 0.4	3.1 ± 0.3	2.5 ± 1.3	4.8 ± 0.3	2.6 ± 0.1	1.4 ± 1.2
125 µg/mL	2.5 ± 1.5	2.5 ± 0.1	0.9 ± 0.3 *	3.8 ± 0.4	1.7 ± 0.1 ****	1.8 ± 0.7
250 µg/mL	1.3 ± 1.4 *	2.1 ±0.2 **	0.9 ± 0.1 *	3.8 ± 0.4	1.2 ± 0.1 ****	1.4 ± 1.3
500 µg/mL	1.1 ± 0.9 **	1.4 ± 0.3 ****	0.5 ± 0.1 **	3.3 ± 1.2	1.3 ± 0.2 ****	0.4 ± 0.3
1000 µg/mL	0.8 ± 0.7 **	1.0 ± 0.9 ****	0.0 ± 0.0 ****	0.8 ± 0.3	0.0 ± 0.0 ****	0.0 ± 0.0 *

Results are reported as the mean ± SD of three experiments. * *p* < 0.05, ** *p* < 0.01, *** *p* < 0.001, **** *p* < 0.0001 vs. control (inhibition = 0) according to two-way ANOVA followed by Tukey’s multiple comparisons test at the significance level of *p* < 0.05.

**Table 3:** Phytotoxic activity of the EOs on *S. arvensis*.

**Germinated Seeds**						
	***E. bicostata***	***E. gigantea***	***E. intertexta***	***E. obliqua***	***E.pauciflora***	***E. tereticornis***
Control (H_2_O)	10.0 ± 0.0	9.3 ± 0.6	9.7 ± 0.6	10.0 ± 0.0	9.7 ± 0.6	9.7 ± 0.6
125 µg/mL	8.3 ± 0.6	8.0 ± 1.0	9.0 ± 1.0	1.7 ± 0.6 ****	7.7 ± 0.6 **	4.7 ± 1.5 ***
250 µg/mL	6.0 ± 2.6 *	4.3 ± 1.2 ****	4.0 ± 0.4 ***	1.7 ± 0.6 ****	5.0 ± 0.0 ****	0.7 ± 0.6 ****
500 µg/mL	0.0 ± 0.0 ****	1.0 ± 0.0 ****	3.0 ± 2.6 ****	0.0 ± 0.0 ****	1.3 ± 0.6 ****	0.0 ± 0.0 ****
1000 µg/mL	0.0 ± 0.0 ****	0.3 ± 0.6 ****	0.0 ± 0.0 ****	0.0 ± 0.0 ****	1.0 ± 0.0 ****	0.0 ± 0.0 ****
**Radical Length (cm)**						
	***E. bicostata***	***E. gigantea***	***E. intertexta***	***E. obliqua***	***E.pauciflora***	***E. tereticornis***
Control (H_2_O)	2.8 ± 0.2	5.6 ± 0.3	3.3 ± 0.6	2.5 ± 0.1	3.7 ± 0.2	3.0 ± 0.7
125 µg/mL	1.1 ± 0.2	1.1 ± 0.3 ****	2.1 ± 0.7	0.7 ± 0.2 ****	1.4 ± 0.2 ****	0.4 ± 0.1 ****
250 µg/mL	1.0 ± 0.4	1.1 ± 0.2 ****	0.5 ± 0.2 ****	0.5 ± 0.1 ****	1.0 ± 0.3 ****	0.2 ± 0.1 ****
500 µg/mL	0.0 ± 0.0 ***	0.5 ± 0.2 ****	0.6 ± 0.5 ****	0.0 ± 0.0 ****	1.6 ± 0.1 ****	0.0 ± 0.0 ****
1000 µg/mL	0.0 ± 0.0 ***	0.1 ± 0.2 ****	0.0 ± 0.0 ****	0.0 ± 0.0 ****	0.7 ± 0.1 ****	0.0 ± 0.0 ****

Results are reported as the mean ± SD of three experiments. * *p* < 0.05, ** *p* < 0.01, *** *p* < 0.001, **** *p* < 0.0001 vs. control (inhibition = 0) according to two-way ANOVA followed by Tukey’s multiple comparisons test at the significance level of *p* < 0.05.

**Table 4:** Phytotoxic activity of the EOs on *L. multiflorum*.

**Germinated Seeds**						
	***E. bicostata***	***E. gigantea***	***E. intertexta***	***E. obliqua***	***E. pauciflora***	***E. tereticornis***
Control (H_2_O)	9.0 ± 1.0	8.3 ± 0.6	10.0 ± 0.0	10.0 ± 0.0	9.7 ± 0.6	7.7 ± 0.6
125 µg/mL	8.7 ± 0.6	9.3 ± 0.6	8.7 ± 0.6	9.3 ± 0.6	9.7 ± 0.6	7.7 ± 1.2
250 µg/mL	8.3 ± 1.5	9.0 ± 0.0	6.3 ± 1.5 *	8.7 ± 0.6	9.0 ± 0.0	8.0 ± 1.0
500 µg/mL	6.0 ± 1.0	7.3 ± 0.6	7.0 ± 0.0	7.7 ± 0.6 *	6.0 ± 1.0 ****	5.3 ± 1.5
1000 µg/mL	4.7 ± 4.2 *	6.7 ± 0.6 *	5.0 ± 1.0 **	6.0 ± 3.0 ****	0.0 ± 0.0 ****	0.0 ± 0.0 ****
**Radical length (cm)**						
	***E. bicostata***	***E. gigantea***	***E. intertexta***	***E. obliqua***	***E. pauciflora***	***E. tereticornis***
Control (H_2_O)	3.6 ± 0.4	5.1 ± 0.5	4.4 ± 0.1	3.0 ± 0.3	4.8 ± 0.2	3.3 ± 0.6
125 µg/mL	3.1 ± 1.0	2.9 ± 0.3 ****	3.3 ± 0.4	1.6 ± 0.2 ***	2.7 ± 0.3 ****	2.2 ± 0.6
250 µg/mL	2.1 ± 0.8	2.7 ± 0.3 ****	2.8 ± 0.2 *	1.3 ± 0.2 ****	2.6 ± 0.2 ****	2.3 ± 0.2
500 µg/mL	2.0 ± 0.1	1.8 ± 0.1 ****	1.8 ± 0.7 ****	0.9 ± 0.2 ****	1.8 ± 0.2 ****	1.2 ± 0.4 ***
1000 µg/mL	1.4 ± 1.5 *	0.6 ± 0.1 ****	0.5 ± 0.1 ****	0.4 ± 0.1 ****	0.7 ± 0.1 ****	0.0 ± 0.0 ****

Results are reported as the mean ± SD of three experiments. * *p* < 0.05, ** *p* < 0.01, *** *p* < 0.001, **** *p* < 0.0001 vs. control (inhibition = 0) according to two-way ANOVA followed by Tukey’s multiple comparisons test at the significance level of *p* < 0.05.

**Table 5:** MIC (µL/mL) of the six *Eucalyptus* EOs necessary to inhibit the growth of *A. baumannii*, *E. coli*, *L. monocytogenes, P. aeruginosa*, and *S. aureus*. Tetracycline (µg/mL) was used as a positive control.

EO	*A. baumannii*	*E. coli*	*L. monocytogenes*	*P. aeruginosa*	*S. aureus*
*E. bicostata*	30 ± 2	25 ± 2	28 ± 2 *	30 ± 2	28 ± 2 ***
*E. gigantea*	25 ± 2 **	23 ± 1	25 ± 2 ***	28 ± 2 **	25 ± 3 ***
*E. intertexta*	35 ± 2	42 ± 1 ***	25 ± 2 ***	28 ± 1 **	28 ± 2 ***
*E. obliqua*	33 ± 2	42 ± 1 ***	28 ± 2 *	30 ± 2	28 ± 3 ***
*E. pauciflora*	25 ± 2 **	35 ± 3 ***	25 ± 2 ***	30 ± 2	35 ± 3
*E. teritcornis*	33 ± 3	28 ± 1	25 ± 2 ***	28 ± 3 **	30 ± 3 ***
Tetracycline	31 ± 2	24 ± 2	33 ± 1	34 ± 1	38 ± 1

The experiments were performed in triplicate and reported as the mean ± SD. * *p* < 0.05, ** *p* < 0.01, *** *p* < 0.0001 vs. tetracycline) according to two-way ANOVA followed by Dunnet’s multiple comparison test at the significance level of *p* < 0.05.

**Table 6:** Percent inhibition of two doses of the EOs on biofilm formation of *A. baumannii*, *E. coli*, *L. monocytogenes, P. aeruginosa*, and *S. aureus* at 0 and 24 h.

Time 0	*A. baumannii*	*E. coli*	*L. monocytogenes*	*P. aeruginosa*	*S. aureus*
*E. bicostata* 10 µL/mL	0.00 ± 0.00	0.00 ± 0.00	54.34 ± 1.25 ****	0.00 ± 0.00	0.00 ± 0.00
*E. bicostata* 20 µL/mL	28.74 ± 1.8 ****	79.61 ± 1.06 ****	65.62 ± 0.31 ****	58.74 ± 2.75 ****	72.55 ± 0.40 ****
*E. gigantea* 10 µL/mL	79.71 ± 0.14 ****	79.22 ± 0.06 ****	82.67 ± 0.10 ****	78.76 ± 0.07 ****	77.51 ± 0.11 ****
*E. gigantea* 20 µL/mL	89.34 ± 0.33 ****	85.70 ± 0.10 ****	85.11 ± 0.16 ****	79.69 ± 0.08 ****	81.61 ± 0.19 ****
*E. intertexta* 10 µL/mL	5.99 ± 0.37 ****	0.00 ± 0.00	0.00 ± 0.00	75.97 * ± 0.17 ****	76.20 ± 0.09 ****
*E. intertexta* 20 µL/mL	63.45 ± 0.21 *	0.00 ±0.00	80.32 ± 0.13 ****	74.70 ± 0.35 ****	76.47 ± 0.18 ****
*E. obliqua* 10 µL/mL	2.96 ± 0.31 ***	0.00 ± 0.00	0.00 ± 0.00	0.00 ± 0.00	0.00 ± 0.00
*E. obliqua* 20 µL/mL	61.50 ± 0.28 ****	0.00 ± 0.00	70.02 ± 0.16 ****	60.21 ± 0.19 ****	70.42 ± 0.30 ****
*E. pauciflora* 10 µL/mL	83.92 ± 0.04 ****	20.54 ± 1.04 ****	0.00 ± 0.00	0.00 ± 0.00	2.02 ± 0.84 ****
*E. pauciflora* 20 µL/mL	86.73 ± 0.02 ****	46.93 ± 0.23 ****	79.47 ± 1.35 ****	13.79 ± 0.83 ****	31.08 ± 5.81 ****
*E. tereticornis* 10 µL/mL	5.89 ± 1.46 ****	0.00 ± 0.00	49.84 ± 1.00 ****	74.17 ± 0.34 ****	5.46 ± 2.30 ****
*E. tereticornis* 20 µL/mL	58.73 ± 0.42 ****	69.57 ± 0.33 ****	83.72 ± 0.45 ****	70.90 ± 0.48 ****	71.80 ± 0.15 ****
**Time 24 h**	*A. baumannii*	*E. coli*	*L. monocytogenes*	*P. aeruginosa*	*S. aureus*
*E. bicostata* 10 µL/mL	62.99 ± 0.91 ****	34.13 ± 0.71 ****	56.63 ± 0.84 ****	51.18 ± 0.45 ****	46.46 ± 0.79 ****
*E. bicostata* 20 µL/mL	73.24 ± 0.29 ****	55.13 ± 1.07 ****	62.82 ± 0.80 ****	85.06 ± 1.92 ****	73.38 ± 0.33 ****
*E. gigantea* 10 µL/mL	40.81 ± 0.66 ****	0.00 ± 0.00	47.01 ± 0.45 ****	66.55 ± 0.84 ****	62.91 ± 0.44 ****
*E. gigantea* 20 µL/mL	51.94 ± 1.15 ****	28.62 ± 0.20 ****	55.44 ± 1.34 ****	69.79 ± 0.57 ****	64.78 ± 0.43 ****
*E. intertexta* 10 µL/mL	32.44 ± 0.68 ****	40.35 ± 1.53 ****	67.75 ± 0.52	56.20 ± 0.99	52.42 ± 0.23 ****
*E. intertexta* 20 µL/mL	27.92 ± 1.01 ****	19.04 ± 0.68 ****	0.00 ± 0.00	36.66 ± 0.77 ****	67.30 ± 0.25 ****
*E. obliqua* 10 µL/mL	23.17 ± 0.96 ****	39.82 ± 1.14 ****	0.00 ± 0.00	7.94 ± 0.74 ****	77.38 ± 0.45 ****
*E. obliqua* 20 µL/mL	34.96 ± 0.68 ****	42.56 ± 0.25 ****	14.52 ± 0.94 ****	29.79 ± 0.55 ****	83.63 ± 0.47 ****
*E. pauciflora* 10 µL/mL	9.71 ± 0.89 ****	28.60 ± 0.74 ****	0.00 ± 0.00	47.97 ± 1.01 ****	49.93 ± 0.54 ****
*E. pauciflora* 20 µL/mL	32.44 ± 1.21 ****	40.35 ± 1.22 ****	67.75 ± 0.32 ****	56.20 ± 0.68 ***	52.42 ± 0.65 ****
*E. tereticornis* 10 µL/mL	10.99 ± 2.21	8.09 ± 3.84 ****	38.86 ± 2.88 ****	13.95 ± 1.33 ****	0.00 ± 0.00
*E. tereticornis* 20 µL/mL	43.88 ± 1.45	33.75 ± 2.06 ****	68.62 ± 2.36 ****	14.10 ± 1.38 ****	24.08 * ± 1.18

The experiments were performed in triplicate, and results were reported as the mean ± SD of three experiments. *: *p* < 0.05, ***: *p* < 0.001, ****: *p* < 0.0001 vs. control (inhibition = 0) according to two-way ANOVA followed by Dunnet’s multiple comparisons test at the significance level of *p* < 0.05.

**Table 7:** Percent inhibition of two doses of the EOs on biofilm metabolic activity of *A. baumannii*, *E. coli*, *L. monocytogenes, P. aeruginosa*, and *S. aureus* at 0 and 24 h.

Time 0	*A. baumannii*	*E. coli*	*L. monocytogenes*	*P. aeruginosa*	*S. aureus*
*E. bicostata* 10 µL/mL	58.01 ± 0.95 ****	0.00 ± 0.00	0.00 ± 0.00	0.00 ± 0.00	54.96 ± 0.48 ****
*E. bicostata* 20 µL/mL	60.88 ± 0.70 ****	0.00 ± 0.00	5.15 ± 0.77 **	34.26 ± 7.33 ****	69.26 ± 1.24 ****
*E. gigantea* 10 µL/mL	83.35 ± 2.78 ****	75.29 ± 1.34 ****	80.04 ± 1.64 ****	79.71 ± 2.04 ****	79.03 ± 1.56 ****
*E. gigantea* 20 µL/mL	85.12 ± 2.88 ****	78.23 ± 1.66 ****	83.22 ± 1.14 ****	81.93 ± 2.19 ****	81.77 ± 1.16 ****
*E. intertexta* 10 µL/mL	0.00 ± 0.00	0.00 ± 0.00	79.14 ± 2.05 ****	75.88 ± 2.59 ****	78.51 ± 1.58 ****
*E. intertexta* 20 µL/mL	69.08 ± 0.35 ****	0.00 ± 0.00	79.48 ± 1.70 ****	78.81 ± 1.78 ****	80.62 ± 0.84 ****
*E. obliqua* 10 µL/mL	52.84 ± 1.06 ****	20.14 ± 1.41 ****	20.67 ± 2.98 ****	1.06 ± 1.17	11.91 ± 1.37 ****
*E. obliqua* 20 µL/mL	57.60 ± 0.98 ****	63.66 ± 1.02 ****	75.41 ± 1.25 ****	74.42 ± 0.33 ****	55.43 ± 1.41 ****
*E. pauciflora* 10 µL/mL	41.05 ± 1.10 ****	0.00 ± 0.00	0.00 ± 0.00	0.00 ± 0.00	57.44 ± 0.81 ****
*E. pauciflora* 20 µL/mL	55.18 ± 0.78 ****	7.70 ± 1.36 ****	44.69 ± 1.82 ****	14.36 ± 2.07 ****	72.03 ± 1.74 ****
*E. tereticornis* 10 µL/mL	0.00 ± 0.00	0.00 ± 0.00	71.13 ± 0.32 ****	0.00 ± 0.00	0.62 ± 1.68
*E. tereticornis* 20 µL/mL	35.63 ± 0.67 ****	62.65 ± 0.40 ****	88.95 ± 0.25 ****	83.06 ± 1.60 ****	85.38 ± 0.18 ****
**Time 24 h**					
*E. bicostata* 10 µL/mL	4.66 ± 3.38 ****	33.52 ± 0.39 ****	29.90 ± 1.09 ****	0.00 ± 0.00	30.88 ± 1.52 ****
*E. bicostata* 20 µL/mL	38.75 ± 1.19 ****	51.34 ± 0.92 ****	54.04 ± 0.88 ****	7.08 ± 0.44 ****	33.22 ± 0.52 ****
*E. gigantea* 10 µL/mL	0.00 ± 0.00	20.59 ± 0.54 ****	0.00 ± 0.00	0.00 ± 0.00	7.95 ± 0.90 ****
*E. gigantea* 20 µL/mL	0.00 ± 0.00	56.68 ± 0.38 ****	0.00 ± 0.00	0.00 ± 0.00	21.38 ± 0.64 ****
*E. intertexta* 10 µL/mL	0.00 ± 0.00	31.89 ± 1.35 ****	0.00 ± 0.00	33.95 ± 0.59 ****	11.46 ± 0.60 ****
*E. intertexta* 20 µL/mL	12.96 ± 1.72 ****	34.32 ± 1.03 ****	0.00 ± 0.00	0.00 ± 0.00	18.77 ± 1.13 ****
*E. obliqua* 10 µL/mL	0.00 ± 0.00	10.32 ± 0.99 ****	0.00 ± 0.00	0.00 ± 0.00	18.43 ± 1.34 ****
*E. obliqua* 20 µL/mL	19.87 ± 1.85 ****	27.08 ± 1.03 ****	0.00 ± 0.00	9.73 ± 0.61 ****	30.54 ± 1.11 ****
*E. pauciflora* 10 µL/mL	0.00 ± 0.00	10.32 ± 0.94 ****	0.00 ± 0.00	0.00 ± 0.00	18.43 ± 1.11 ****
*E. pauciflora* 20 µL/mL	88.39 ± 1.07 ****	27.08 ± 0.82 ****	0.00 ± 0.00	9.73 ± 0.71 ****	30.54 ± 0.81 ****
*E. tereticornis* 10 µL/mL	22.79 ± 1.12 ****	0.00 ± 0.00	26.68 ± 1.03 ****	44.15 ± 0.12 ****	25.62 ± 0.35 ****
*E. tereticornis* 20 µL/mL	34.69 ± 0.23 ****	65.92 ± 0.87 ****	29.26 ± 0.06 ****	45.63 ± 0.28 ****	64.24 ± 1.15 ****

The experiments were performed in triplicate, and results were reported as the mean ± SD of three experiments. **: *p* < 0.01, ****: *p* < 0.0001 vs. control (inhibition = 0) according to two-way ANOVA followed by Dunnet’s multiple comparisons test at the significance level of *p* < 0.05.

**Table 8:** Data on plant material, yields, place of origin and climatic conditions.

	Arboretum (Governorate)	Harvest Period	Bioclimatic Conditions	Yield (%)
*E. bicostata*	Choucha (Bizerte)	March 2021	Upper humid	1.40
*E. gigantea*	Zerniza (Bizerte)	July 2021	Upper humid	0.20
*E. intertexta*	Djebel Manasour (Zaghouen)	May 2021	Upper and middle semi-arid	0.55
*E. obliqua*	HenchirNaam (Siliana)	April 2021	Upper and middle semi-arid	3.11
*E. pauciflora*	Zerniza (Bizerte)	July 2021	Upper humid	0.10
*E. tereticornis*	Zerniza (Bizerte)	July 2021	Upper humid	0.03

## Data Availability

The data presented in this study are available on request from the corresponding author.
